# Supplementary material for: The cryo-EM structure of the acid activatable pore-forming immune effector Macrophage-expressed gene 1
Source: Nat Commun. 2019 Sep 19;10:4288. doi: 10.1038/s41467-019-12279-2 (PMC6753088; doi:10.1038/s41467-019-12279-2)
Supplement: Supplementary file 1 — Supplementary Information [file 41467_2019_12279_MOESM1_ESM.pdf]

The structure of Macrophage-Expressed Gene 1 a phagolysosome immune effector that is activated upon acidification

Pang, Bayly-Jones *et al*.

SUPPLEMENTARY INFORMATION

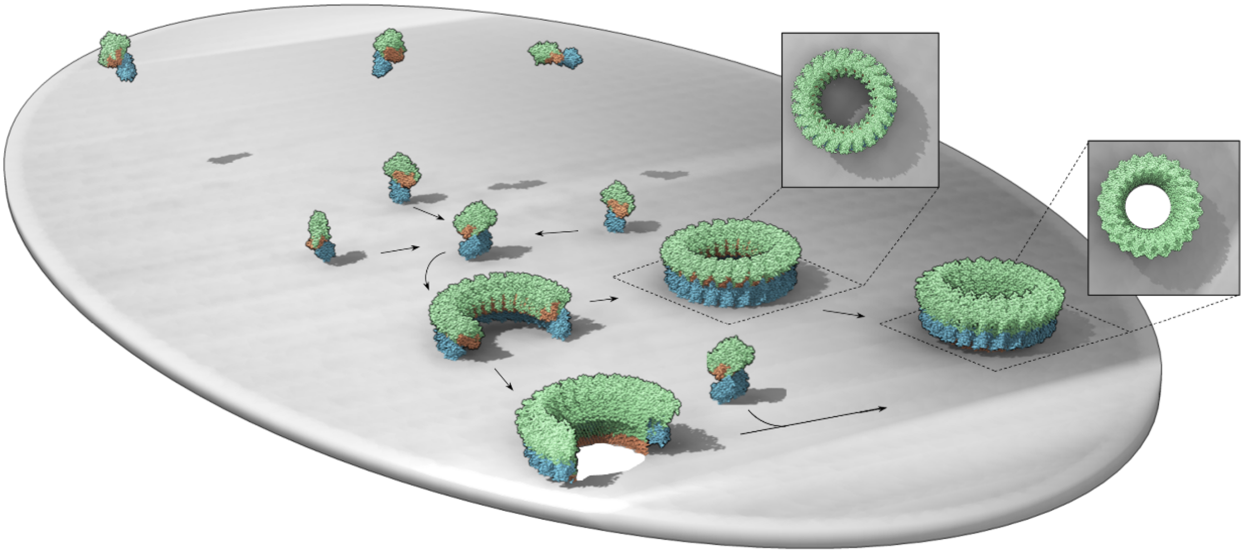

Supplementary Figure 1. Simple illustration of the canonical CDC mechanism of pore formation. Initially freely diffusing soluble subunits recognise and bind to target membranes by employing a target recognition domain. Membrane bound monomers undergo two-dimensional diffusion along the surface of the membrane assembling into larger oligomers that have yet to insert into the membrane i.e. prepores or arcs. These prepores (complete or incomplete) can then undergo a conformation change whereby the TMH regions unfurl and insert into the membrane forming a giant (complete or incomplete) β-barrel pore. MACPFs (e.g., MAC and perforin) have shown variations of this mechanism, with oligomerisation largely coinciding with membrane insertion[1,2]. Insets represent views from above.

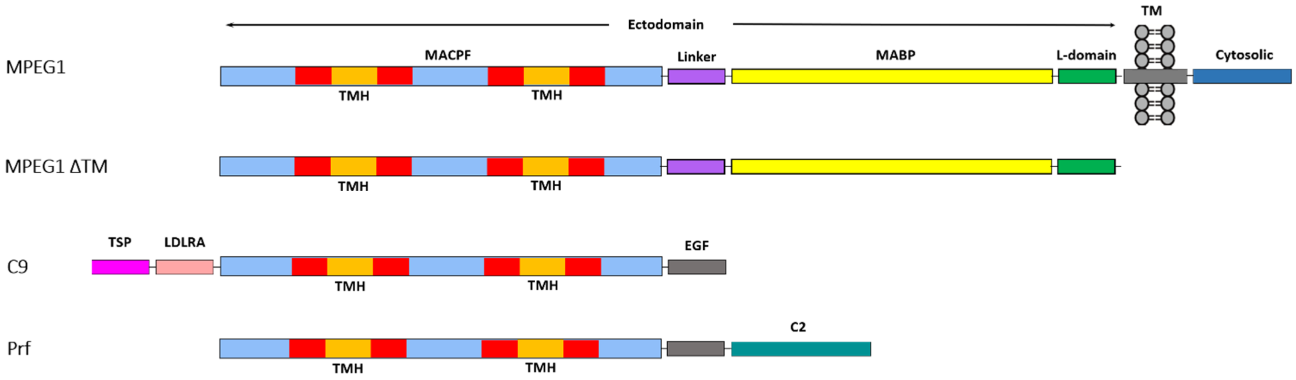

Supplementary Figure 2. Schematic of MPEG1 domain architecture. MPEG1, complement C9 and perforin share a homologous MACPF domain, however only MPEG1 possesses a transmembrane tether and cytosolic region (missing in our construct, depicted here as MPEG1 ΔTM). The proposed orientation of MPEG1 in the vesicular compartment places the pore forming domain in the acidic lumen of the phagosome, while the cytosolic region is exposed in the cytoplasm.

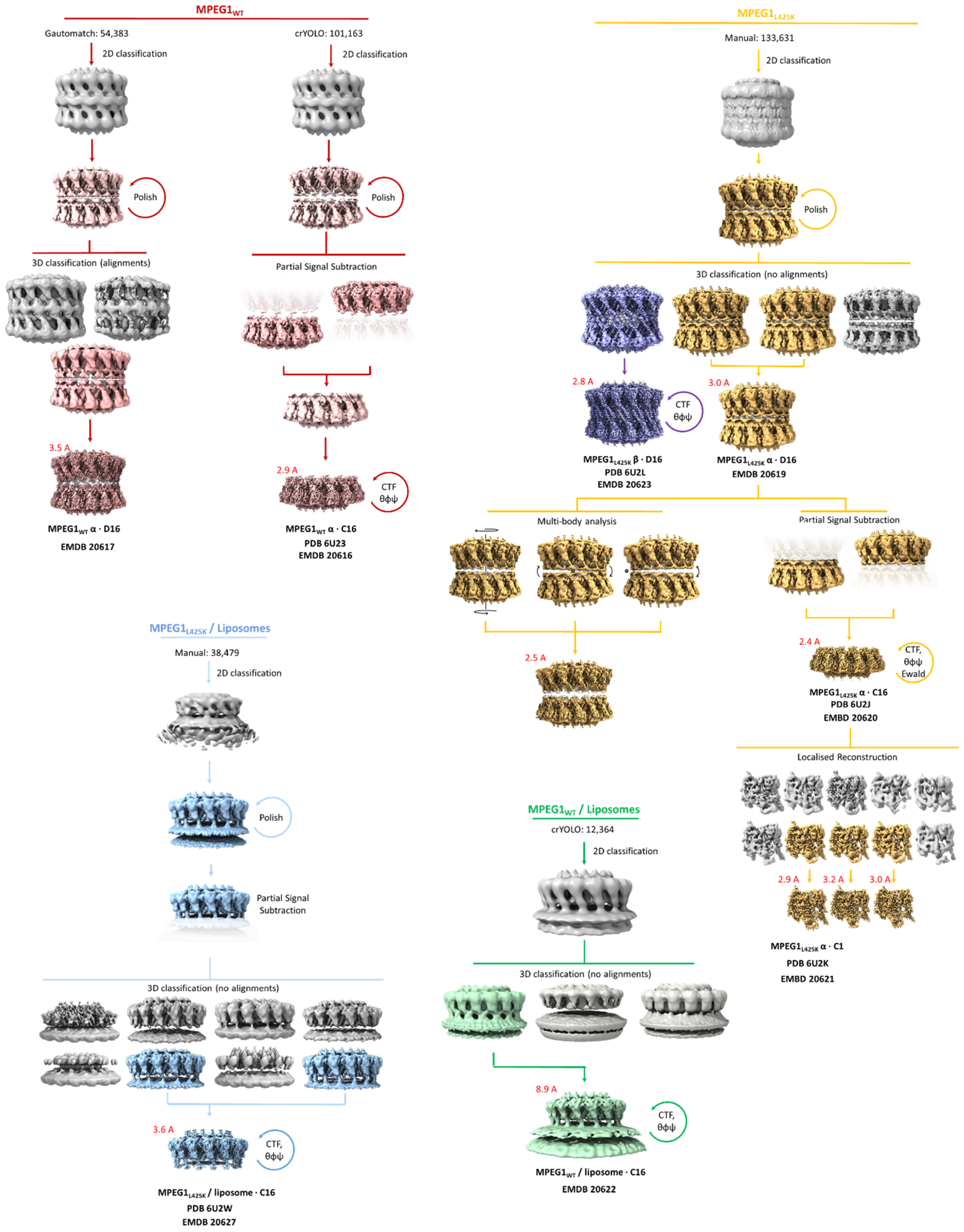

Supplementary Figure 3. Summarised overview of the analysis and workflow for all four cryo-EM data sets. Two data sets of the soluble head-to-head assembly were collected, namely MPEG1$_{WT}$ (rose) and MPEG1$_{L425K}$ (yellow/purple). Additionally, two data sets of the lipid-bound MPEG1 prepore assembly were obtained, referred to as MPEG1$_{WT}$ / liposome (green) and MPEG1$_{L425K}$ / liposome (blue).

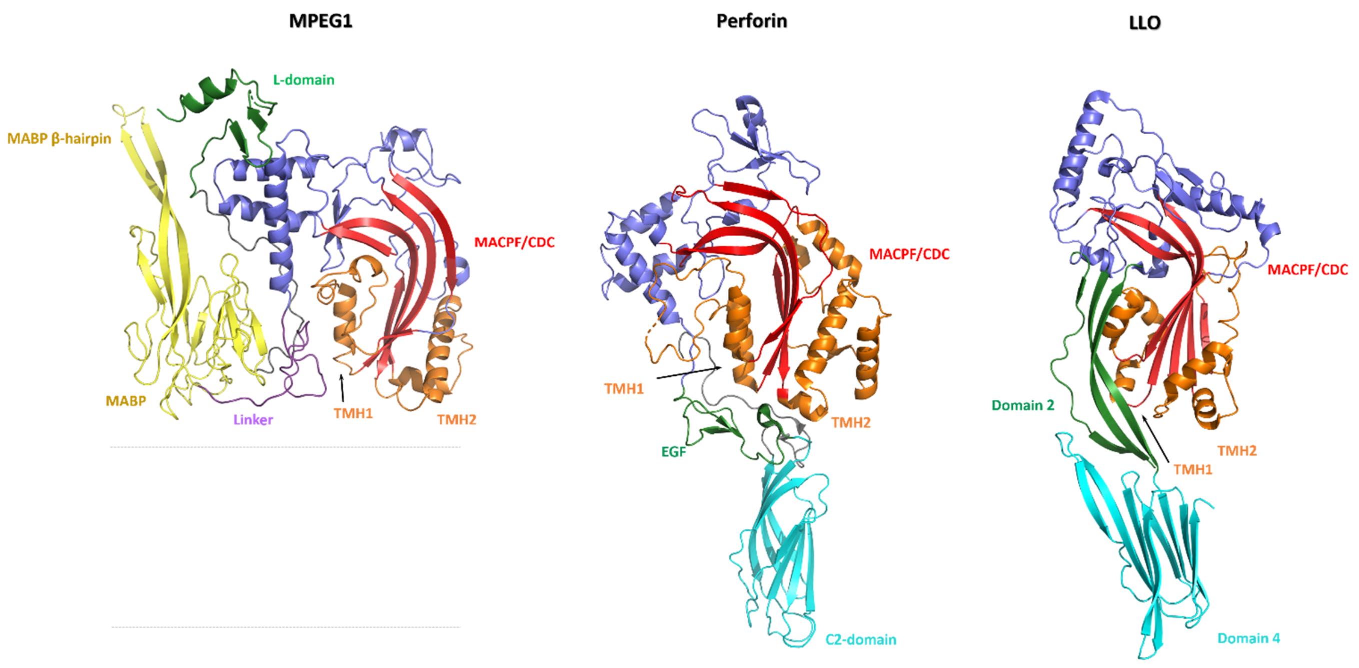

Supplementary Figure 4. Structural comparison between MPEG1, lymphocyte perforin and lysteriolysin O (LLO). The pore forming MACPF/CDC domain is coloured blue, with the central β-sheet in red and TMH1/2 in orange. Notably in these exemplars (perforin [PDB 3NSJ], LLO [PDB 4CDB]), the MACPF/CDC domain is oriented in the same direction as the ancillary membrane targeting domain, while the ancillary membrane binding domain of MPEG1 is inverted relative to the inserting direction of the MACPF domain (MPEG1; yellow. Perforin C2 domain and LLO domain 4; cyan). The shelf domain, which in CDCs facilitates collapse during pore formation, is coloured green.

a

b

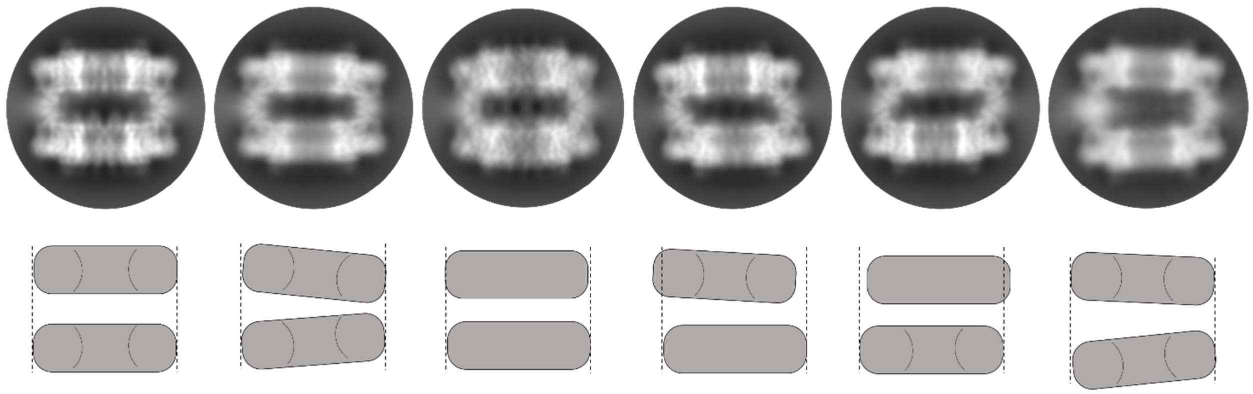

Supplementary Figure 5. Conformational variability of wild type MPEG1 as observed by 2D class averages from cryo-electron microscopy. a) Comparison between 2D class averages of vitrified MPEG1 WT reveals each 16-mer ring can adopt a range of positions relative to one another and hence do not adopt one single, well defined conformational state. **b**) Below each 2D class average is a simplified cartoon model to depict the variation between each class.

a b

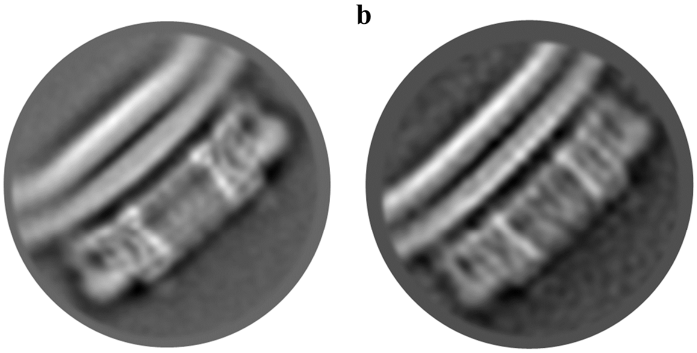

MPEG1$_{L425K}$ POPC:POPS

MPEG1$_{L425K}$ *E. coli* lipid

Supplementary Figure 6. Comparison between 2D class averages of vitrified MPEG1 (L425K) bound to liposomes of two different lipid composition. a) Side view of MPEG1 bound to POPC/POPS liposomes, and **b**) corresponding view of MPEG1 bound to liposomes of *E. coli* total lipid extract.

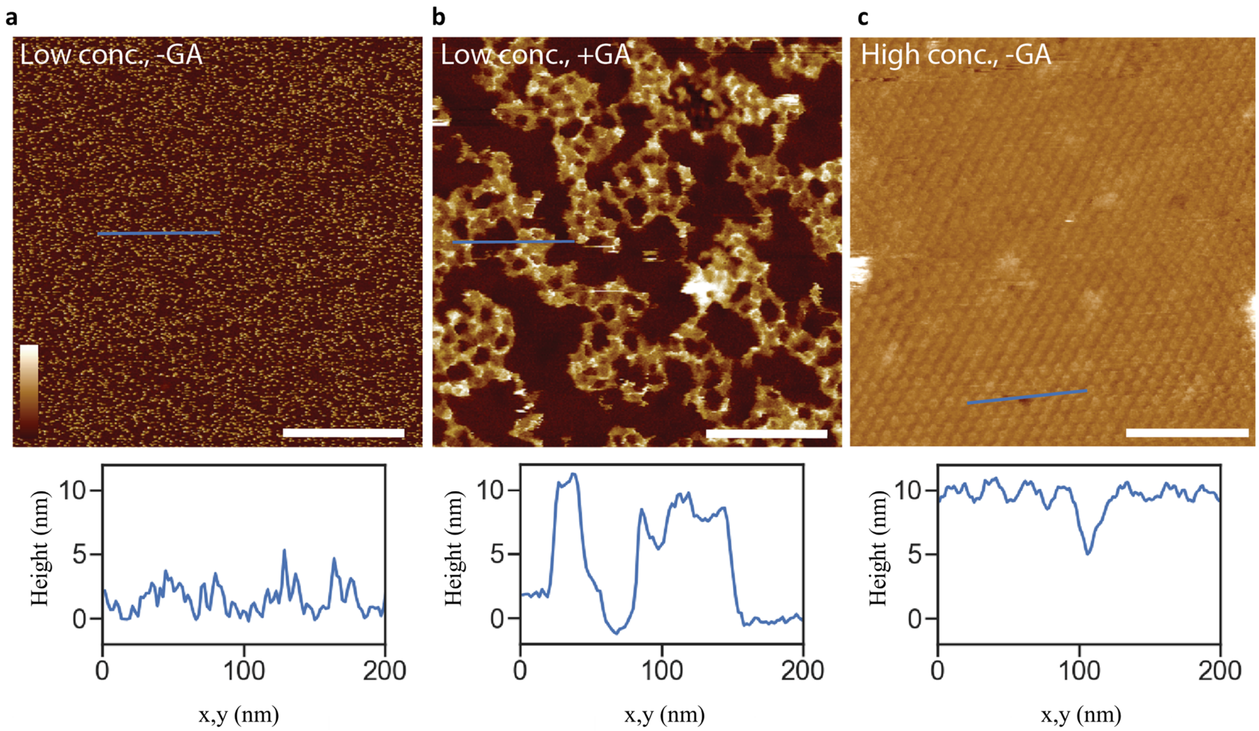

Supplementary Figure 7. Atomic force microscopy images of MPEG1 on supported lipid bilayers consisting of POPC:POPS (1:1 % mol). a) Without (-GA) and **b)** with (+GA) glutaraldehyde fixation, at neutral pH. Glutaraldehyde fixation appears to create more heterogenous sample, perhaps due to cross-linking between MPEG1 and the membrane. **c)** Increasing the concentration of MPEG1 results in a high-density, hexagonal packing on the supported bilayer. The dynamic motion observed in **a)** is therefore reduced such that the MPEG1 assemblies can be well resolved without glutaraldehyde fixation. Shown below each AFM image are 1D height profiles extracted from selected regions (blue lines) where both membrane and MPEG1 prepores are observed. Scale bar: 200 nm. Colour (height) scale: 16 nm.

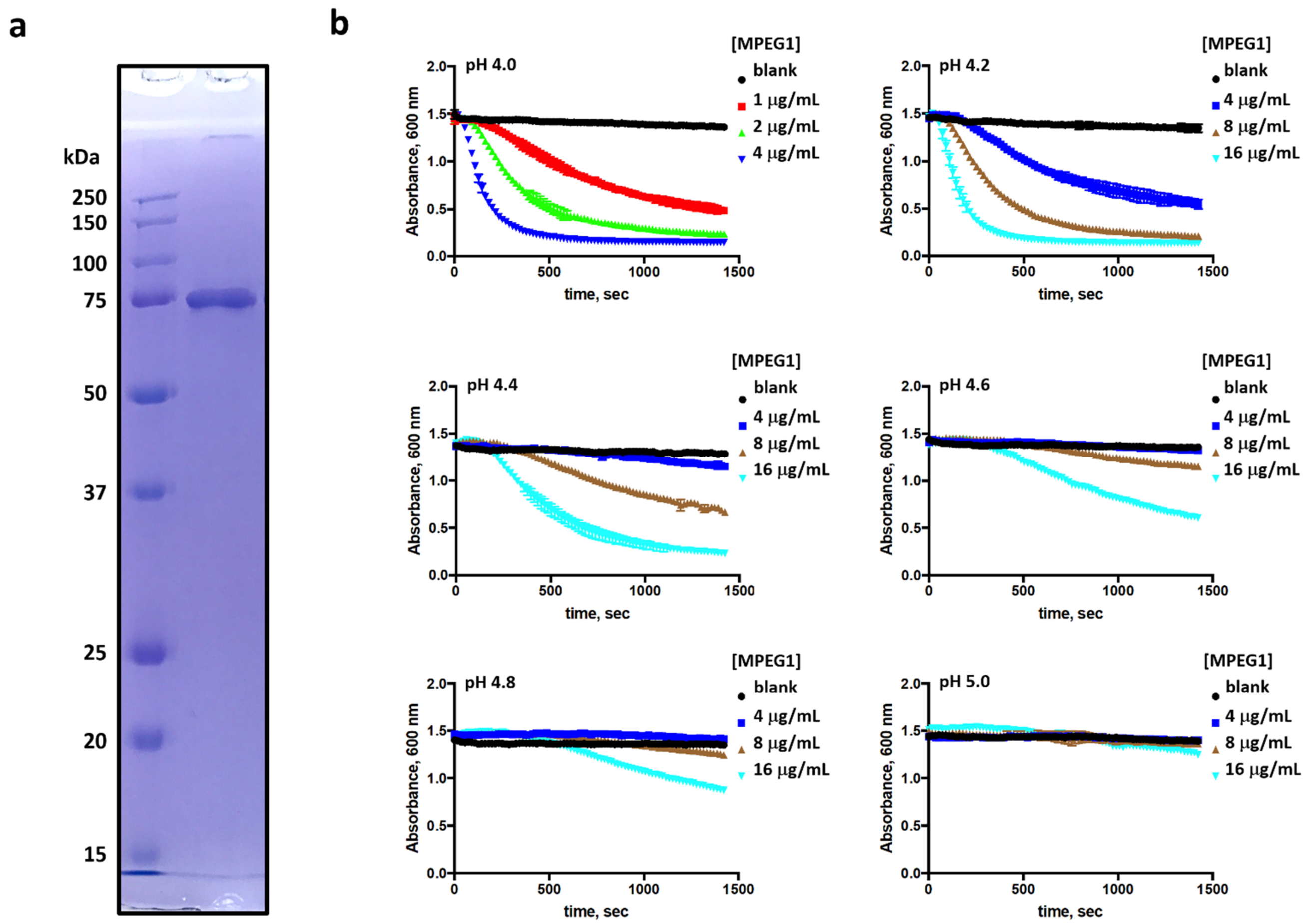

Supplementary Figure 8. Recombinant purified MPEG1 and corresponding acid induced lytic activity. a) 15% (v/v) SDS-PAGE of recombinant human MPEG1 showing a single species that migrates at an apparent molecular weight of ~75 kDa. **b**) Examples of the activity curves of a typical RBC lysis assay in the presence of various concentrations of MPEG1 at different pH. The assays were done in duplicates (n=2). Each data point is shown as an average with its associated standard deviation. Error is plotted for all data points.

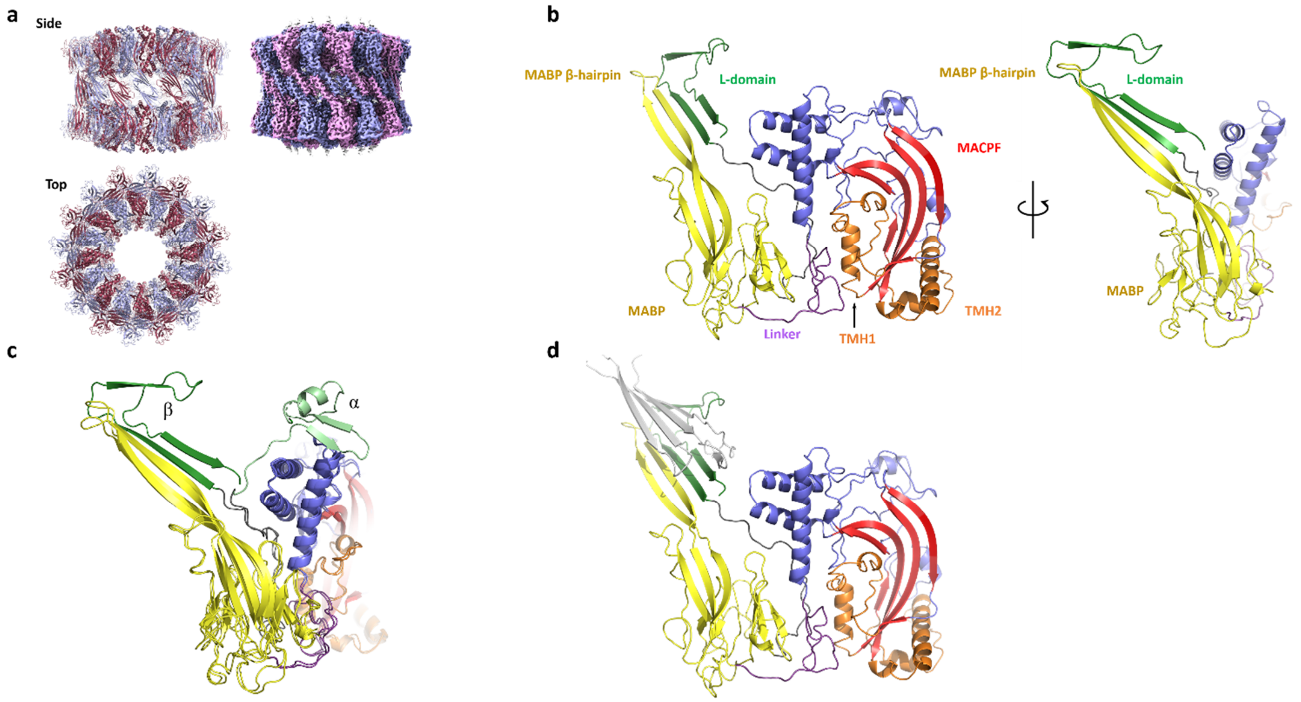

Supplementary Figure 9. Subpopulation of MPEG1$_{L425K}$ observed by cryo-EM adopts an alternative β-conformation. a) Structure (left) and cryo-EM reconstruction (right) of the MPEG1$_{L425K}$ β-conformation assembly shown in alternating colours (EMDB 20623, PDB 6U2L). **b)** The L-domain adopts a unique conformation resulting in a four stranded β-sheet extending from the original β-hairpin of the MABP domain. **c)** Structural superposition of the α- and β-conformations. Rearrangement of the L-domain from the α- to the β-conformation causes inter-subunit interactions with adjacent subunits to break, the β-conformation results in new **d)** in trans interactions between opposing 16-mer rings of the soluble head-to-head assembly (shown here partially in grey).

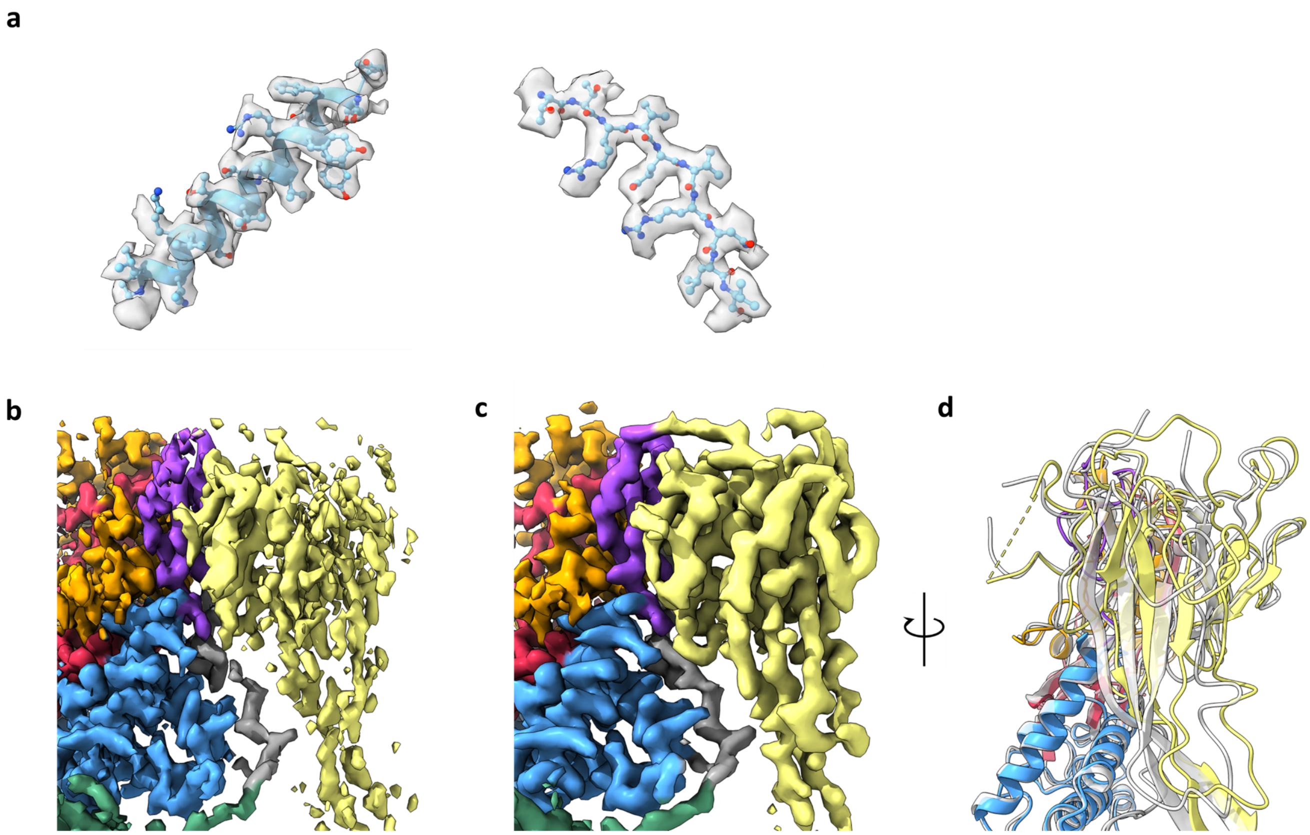

Supplementary Figure 10. Conformational flexibility in the MABP domain. a) Select regions of electron density at the central core region of the MACPF domain from the C16 reconstruction of MPEG1$_{L425K}$ (EMDB 20620) showing high resolution features. **b**) The same reconstruction has poor, anisotropic resolution in the peripheral MABP domain, however after localised reconstruction **c**) the intramolecular heterogeneity can be resolved (EMDB 20621). **d**) Structural superposition of two discrete conformations of the MABP domain.

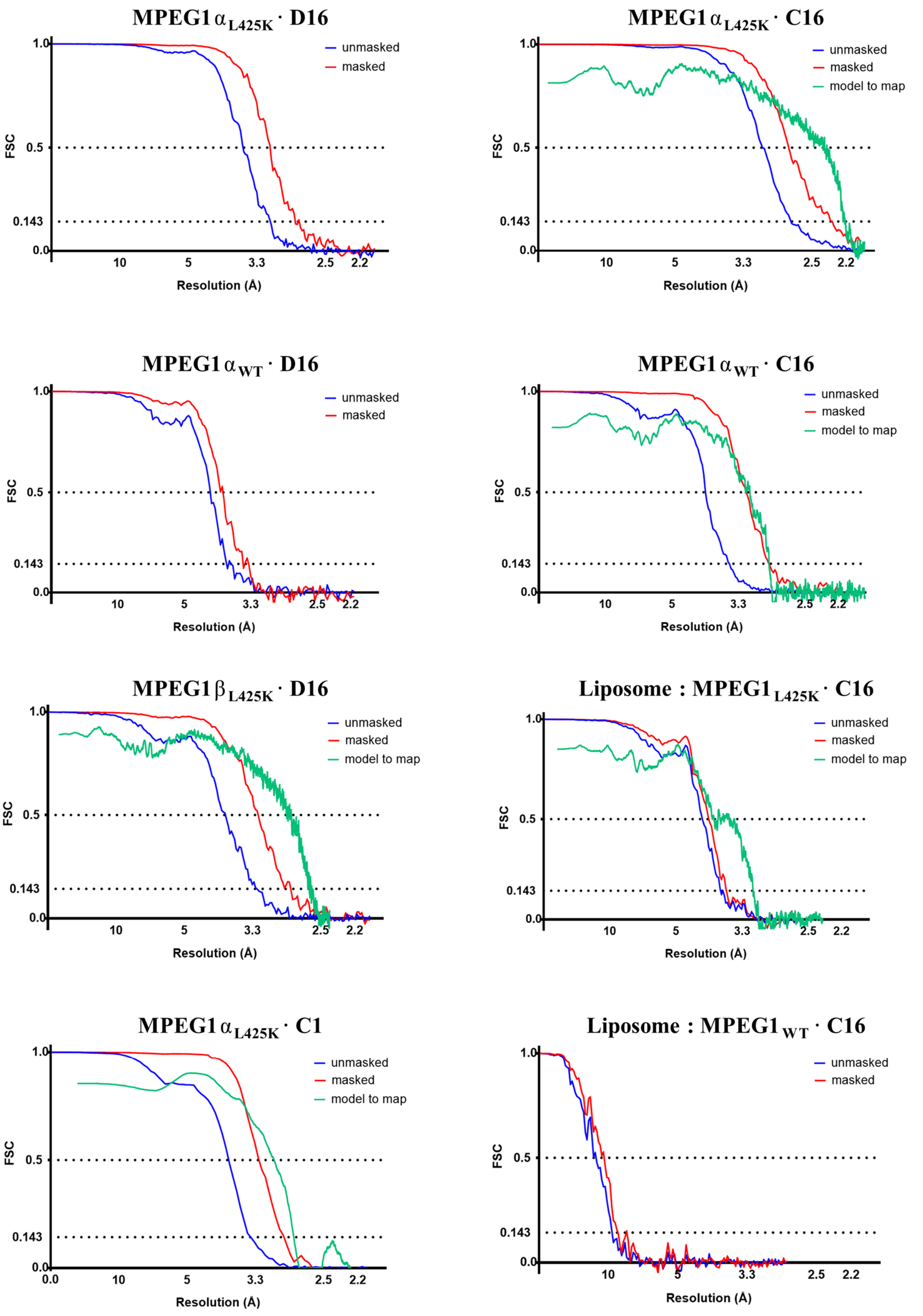

Supplementary Figure 11. Gold-standard Fourier shell correlation (FSC) global resolution estimate. Two FSC criteria are indicated at 0.5 and 0.143 thresholds by dotted lines. The FSC of masked half-maps (red), unmasked half-maps (blue) and model to half-map (green) are plotted (only for maps where atomic models were generated).

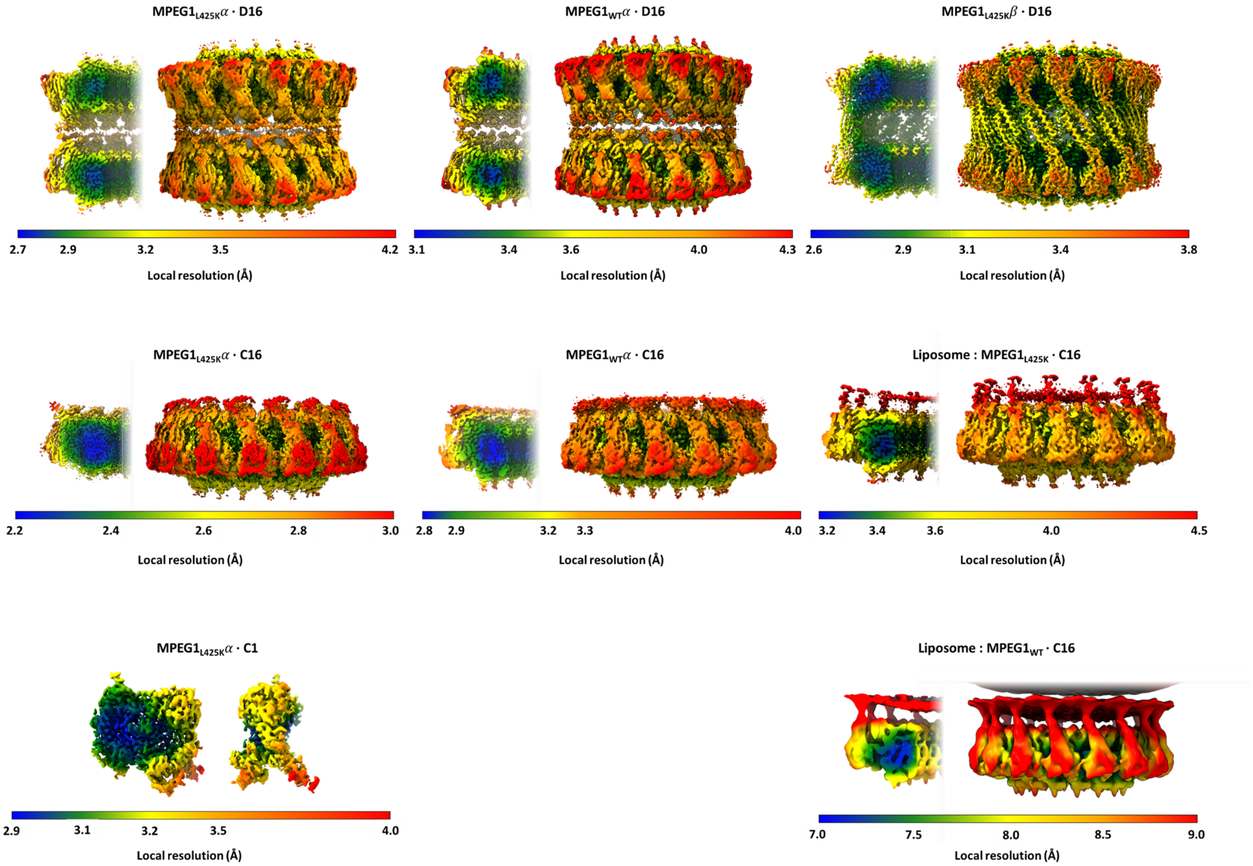

Supplementary Figure 12. Local variation in resolution by gold-standard Fourier shell correlation (FSC) at 0.143. Local resolution ranges in Ångström (Å) are displayed as a colour gradient (blue to red) on the locally filtered, sharpened maps. Colour scales are provided below each reconstruction.

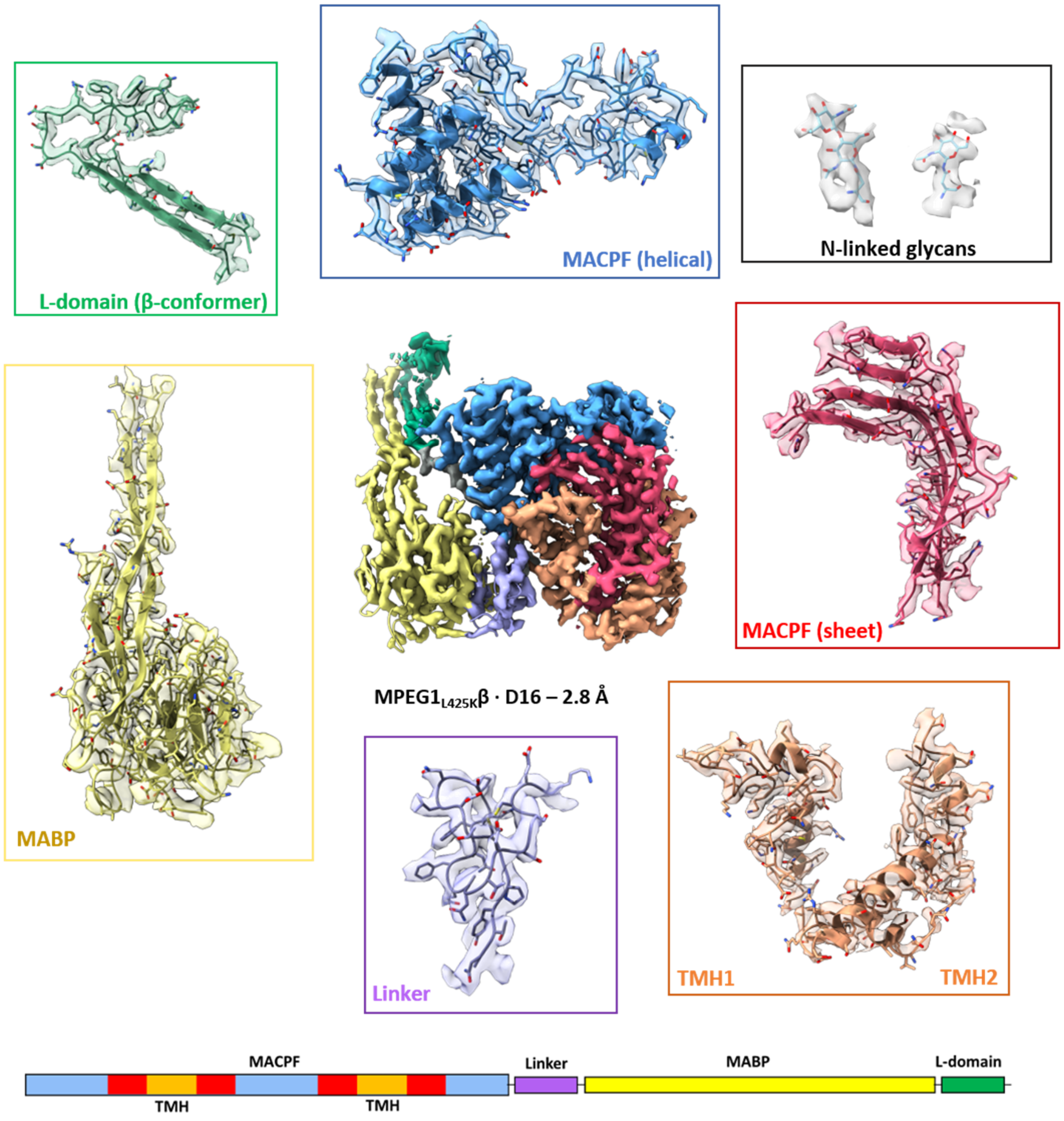

Supplementary Figure 13: Qualitative comparison between atomic models and cryo-EM reconstruction of the D16 MPEG1$_{L425K}$ β reconstruction. A single monomer of MPEG1 is shown (centre) and corresponding domain schematic (below). Extracted regions of electron density corresponding to each domain are boxed and labelled, electron density is shown as a transparent surface and atomic model cartoon and side chains displayed beneath. All boxes, labels and surfaces are coloured consistently with Fig. 1. and the domain schematic.

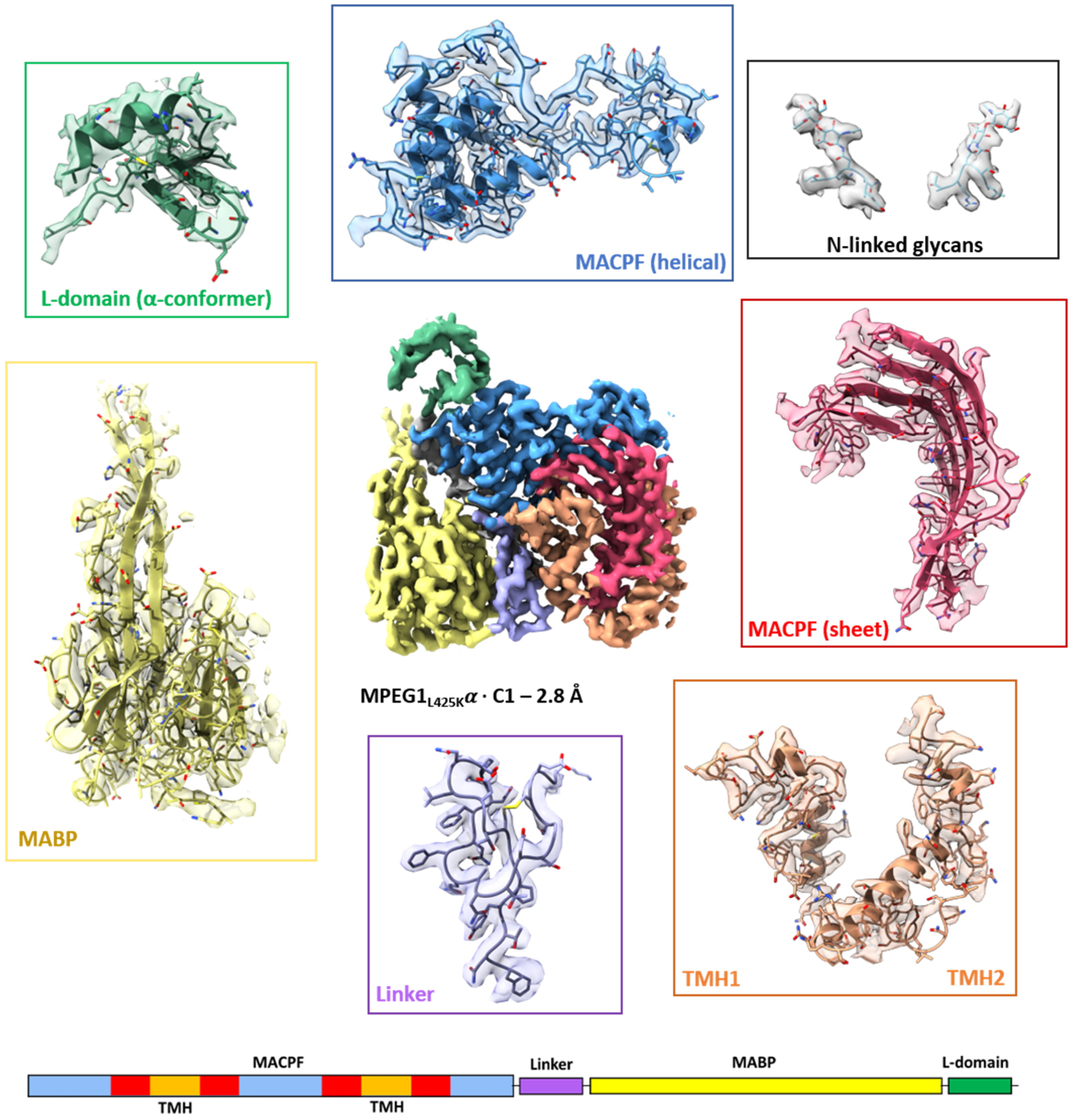

Supplementary Figure 14. Qualitative comparison between atomic models and cryo-EM reconstruction of the C1 MPEG1$_{L425K}$ α reconstruction. Colouring of content as shown in Supp. Fig. 13.

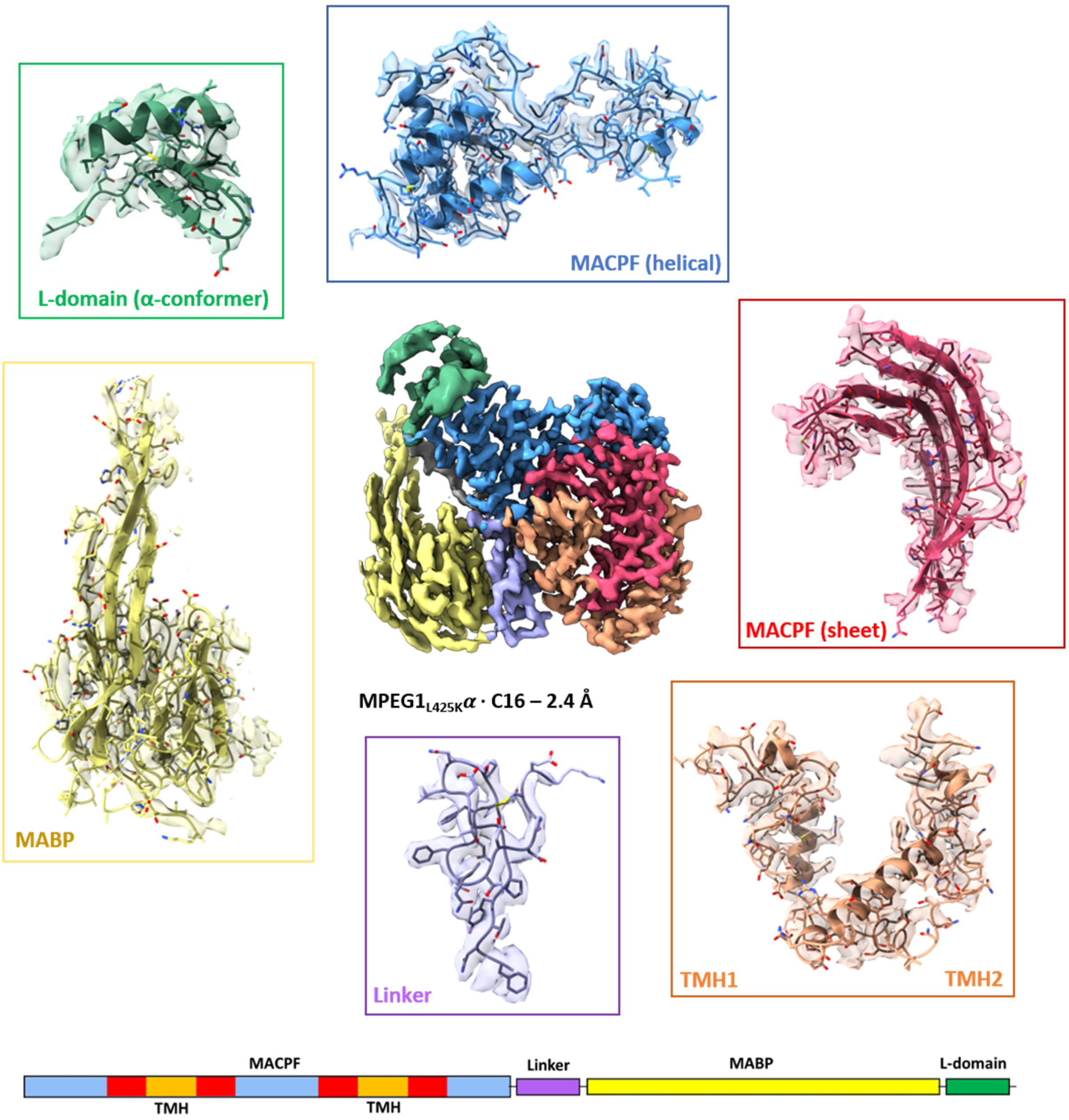

Supplementary Figure 15. Qualitative comparison between atomic models and cryo-EM reconstruction of the C16 MPEG1$_{L425K}$ α reconstruction. Colouring of content as shown in Supp. Fig. 13.

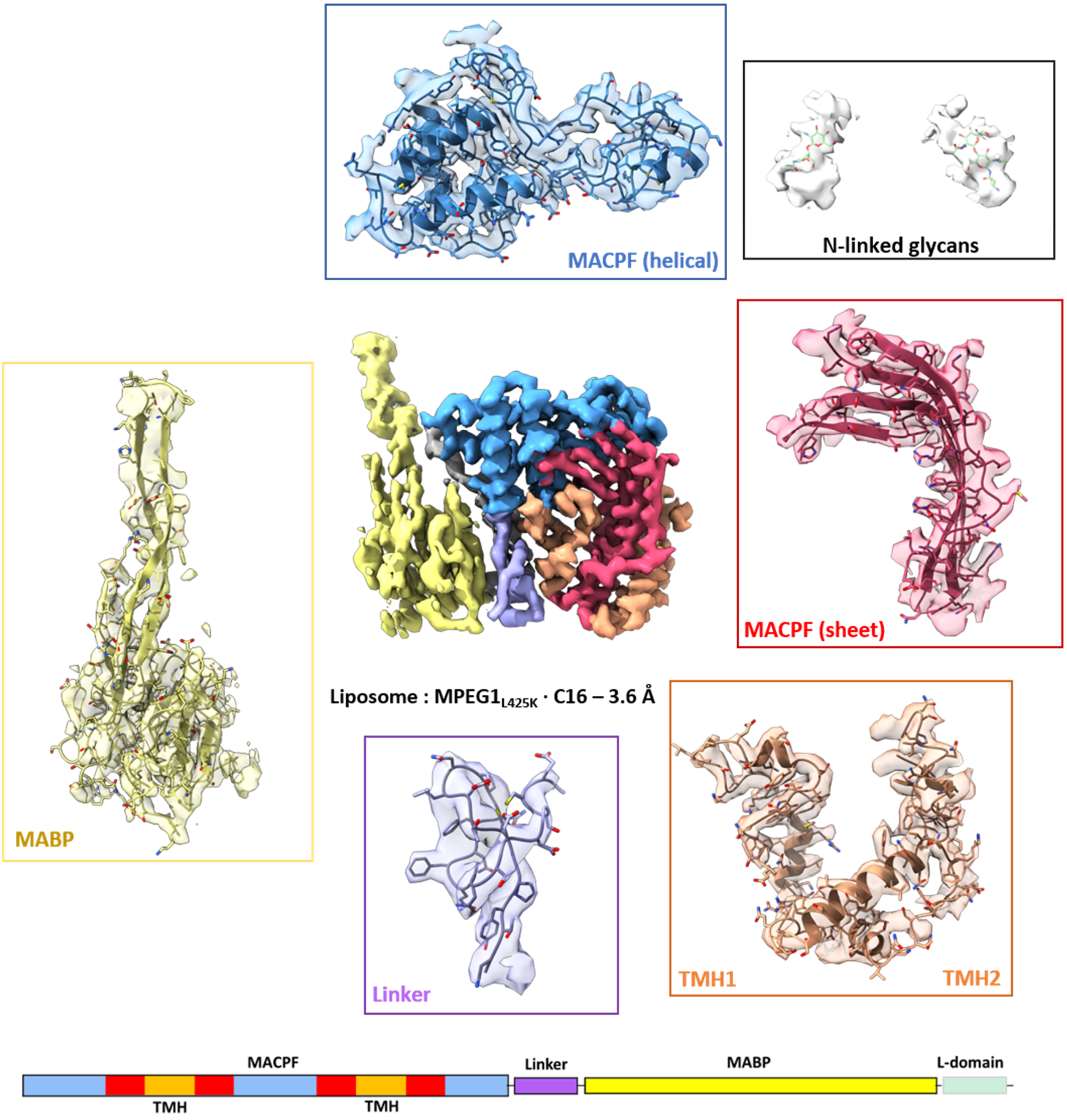

Supplementary Figure 16. Qualitative comparison between atomic models and cryo-EM reconstruction of the C16 MPEG1$_{L425K}$ liposome bound reconstruction. Colouring and content as shown in Supp. Fig. 13. The L-domain becomes flexible or disordered upon MPEG1 membrane binding and, hence, it is unresolved. It is intentionally not shown and this region in the domain schematic is transparent to reflect its absence.

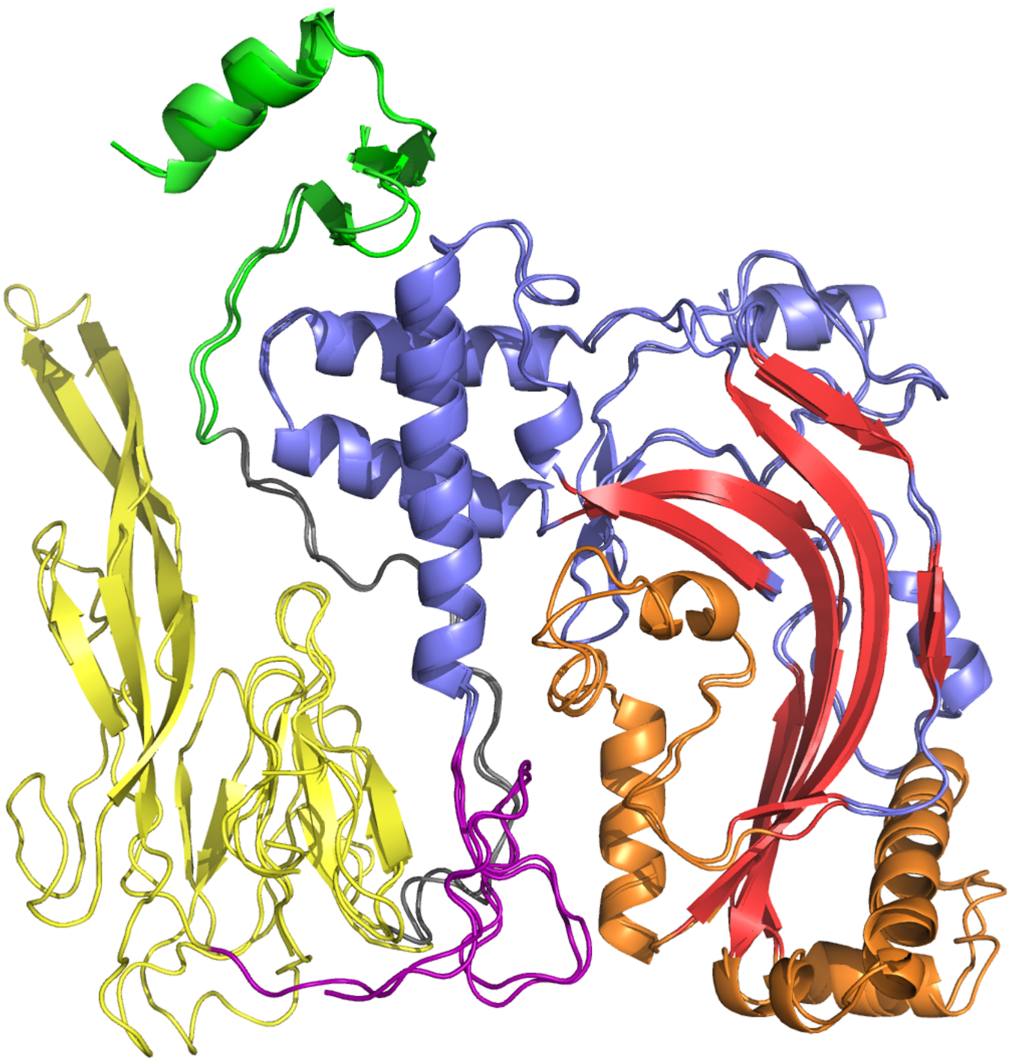

Supplementary Figure 17. Structural superposition of wild type (PDB 6U23) and L425K (PDB 6U2J) MPEG1 monomers isolated from D16 head-to-head assemblies (α-conformation).

Supplementary Table 1. Contacts (≤ 4 Å) between adjacent MPEG1 monomers (1 and 2) in soluble assembly (PDB6U23). The interface contacts were generated using CCP4 (CONTACT)[3] and PISA[4]. The contacts found around the central lumen of the β-barrel are shaded in red, and the in trans subunit interactions between the L-domain and the adjacent MABP β-hairpin are shaded in green.

Monomer 1		Monomer 2		H/S*	Distance, Å
Leu21	C	Gln15	E1		3.76
Thr55	OG1	Lys18	NZ	H	3.69
Glu57	O	Val25	CB		3.93
		Val25	O		3.42
Glu57	CG	Glu27	CA		3.90
		Glu27	CB		3.72
Asp58	CG	Glu27	OE1		3.92
Asp58	OD1	Glu27	CD		3.81
		Glu27	OE1		2.91
Gly59	CA	Lys18	NZ		3.56
Phe68	CZ	Gly43	N		3.66
Ile70	CG2	Asp41	OD2		3.67
Ile70	CD1	Asp41	O		3.72
		Asp41	CG		3.79
		Asp41	OD1		3.19
		Asp41	OD2		3.98
Pro71	O	Val284	CG1		3.76
Pro71	CB	Glu27	OE1		3.63
Pro71	CG	Glu27	O		3.90
		Glu27	OE1		3.76
Gln72	O	Val284	CG1		3.61
		Ile261	O		3.14
Lys73	C	Ile261	O		3.42
Lys73	O	Ile261	C		3.53
		Ile261	O		2.96
Lys73	CB	Val284	CG1		3.92
		Ile261	O		3.66
Lys73	CG	Val284	CG1		3.83
Lys73	NZ	His282	ND1		3.41
		Glu27	OE1	S	3.98
		Glu27	OE2	S	3.79
Gln74	CA	Ser260	O		3.42
Gln74	C	Ser260	O		3.71
Gln74	CG	Ser260	O		3.90
Gln74	NE2	Gln259	OE1	H	3.07
Ser75	N	Ser260	O	H	3.10
Ser75	O	Val258	O		3.58
		Ser260	N	H	3.32
		Gln259	CA		3.28

		Gln259	C		3.82
		Gln259	CB		3.76
Ser75	CB	Ser260	OG		3.52
Ser75	OG	Phe266	N		3.98
Asn76	C	Val258	O		3.83
Asn76	OD1	Gln259	CB		3.43
		Gln259	CD		3.91
		Gln259	OE1		3.06
Leu77	N	Val258	O	H	3.23
Leu77	CA	Val258	O		3.78
Leu77	O	Arg257	CB		3.53
		Val258	O		3.75
		Arg257	CA		3.28
		Arg257	C		3.64
		Val258	N	H	3.01
Leu77	CB	Val258	O		3.39
Leu77	CD2	Pro268	CD		3.97
		Phe266	CD2		3.85
		Phe266	CE2		3.92
		Pro268	CG		3.93
Glu78	CA	Ser256	O		3.91
Glu78	C	Ser256	O		3.95
Glu78	CD	Asn255	ND2		3.55
		Ser256	O		3.57
Glu78	OE1	Arg257	CB		3.30
		Ser256	O		3.95
		Arg257	CG		3.97
Glu78	OE2	Asn255	ND2	H	2.30
		Ser256	O		3.24
		Ser256	C		4.00
		Asn255	CG		3.27
		Asn255	OD1		3.56
Met79	N	Ser256	O	H	3.05
Met79	CA	Ser256	O		3.85
Met79	CB	Ser256	O		3.68
Met79	SD	Ser256	O		3.86
Met79	CE	Val258	CG2		3.89
Asn80	CB	Asn255	ND2		3.50
Asn80	CG	Asn255	ND2		3.88
Asn80	OD1	Asn255	ND2	H	3.50
		Asn255	N		3.99
		Asn255	CA		3.31
		Asn255	C		3.54
		Ser256	N	H	3.32
Thr100	O	Pro573	CG		3.83
Leu102	CD1	Arg575	NE		4.00

Ser103	C	Ser468	O		3.67
Ser103	O	Lys469	CA		3.86
		Lys469	C		3.97
Leu104	N	Ser468	O		3.28
Leu104	CA	Ser468	O		3.52
		Gln478	NE2		3.49
Leu104	C	Gln478	NE2		3.35
Leu104	O	Gln478	CD		3.16
		Gln478	OE1		3.37
		Gln478	NE2	H	2.52
Leu104	CB	Ser468	O		3.57
Leu104	CG	Gln478	OE1		3.49
		Gln478	NE2		3.74
Leu104	CD1	Ser468	CA		3.90
		Ser468	OG		3.69
Leu104	CD2	Gln478	CD		3.99
		Gln478	OE1		3.15
Lys107	CD	Asn476	OD1		3.82
Lys143	NZ	Asp41	O	H	3.67
		Met42	CB		3.55
Pro146	CB	Glu157	OE2		3.87
		Asn183	CG		3.91
		Asn183	ND2		3.83
Pro146	CG	Asn183	CB		3.79
		Asn183	CG		3.83
Ser217	OG	Arg253	C		3.92
		Arg253	O	H	2.83
		Thr254	CA		3.77
Leu225	CD1	Phe235	CZ		3.68
Gln228	OE1	Phe235	CD1		3.39
		Phe235	CE1		3.15
		Phe235	CZ		3.62
Asn229	OD1	Phe235	CE2		3.91
Phe233	CE1	Glu236	OE2		3.98
Phe233	CZ	Glu236	OE2		3.80
Leu272	CD2	Pro268	CD		3.84
Gln273	CB	Tyr267	CD2		3.96
		Tyr267	CE2		3.78
		Tyr267	CZ		3.95
Gln273	CG	Tyr267	CE2		3.96
		Tyr267	CZ		3.70
		Tyr267	OH		3.77
Gln273	CD	Tyr267	CZ		3.92
		Tyr267	OH		3.60
Gln273	NE2	Tyr267	OH		3.65
Gln276	CD	Pro265	CB		3.92

		Phe266	O		3.55
Gln276	OE1	Tyr267	CD1		3.81
		Tyr267	CB		3.70
		Pro265	C		3.96
		Pro265	O		3.87
		Pro265	CB		3.73
		Phe266	C		3.69
		Phe266	O		3.16
Gln276	NE2	Phe266	O	H	3.14
Pro308	CB	Phe436	CG		3.98
		Phe436	CD2		3.64
		Phe436	CE2		3.62
		Phe436	CZ		3.94
Lys311	NZ	Pro583	C		3.97
		Pro583	O	H	2.87
		Leu584	CA		3.89
		Leu584	C		3.97
Thr347	CG2	Ala477	CB		3.83
Asp348	N	Ala477	CB		3.98
Asp348	O	Ala477	CB		3.45
Asp348	CB	Thr475	O		3.37
Asp348	CG	Thr475	O		3.56
Asp348	OD2	Thr475	O		3.07
Asp349	O	Cys480	O		3.92
Gly350	O	Ala482	CB		3.96
Glu353	CD	Arg506	NH2		3.98
Glu353	OE1	Arg506	NE	S	3.95
		Arg506	CZ		3.93
		Arg506	NH2	S	3.83
Glu353	OE2	Arg506	NE	S	3.60
		Arg506	CZ		3.78
		Arg506	NH2	S	3.30
Asn591	N	Thr430	O	H	3.34
Asn591	CA	Thr430	O		3.77
Asn591	C	Cys432	N		3.95
Asn591	O	Thr430	O		3.55
		Val431	CA		3.40
		Val431	C		3.42
		Cys432	N	H	2.74
		Cys432	CA		3.58
		Cys432	CB		3.49
		Cys432	SG		3.95
		Cys432	O		3.98
Asn591	CB	Thr430	O		3.33
Thr592	CA	Cys432	O		3.67
Thr592	CG2	Leu416	CD1		4.00

Val593	N	Cys432	O	H	3.46
Ile594	O	Asp434	OD1		3.29
		Glu433	CB		3.62
		Glu433	CA		3.60
		Asp434	N	H	3.80
Ile594	CG2	Glu433	CB		3.64
Val595	CA	Asp434	OD1		3.44
Val595	CB	Asp434	OD1		3.72
Val595	CG1	Asp434	OD1		3.92
Val595	CG2	Asp434	CG		3.97
		Asp434	OD1		3.28
		Asp434	OD2		3.85
Thr596	N	Asp434	O	H	3.23
Thr596	C	Asp434	O		3.93
Thr596	O	Phe436	CD2		3.24
		Phe436	CE2		3.95
		Asp434	C		3.95
		Asp434	O		3.09
		Val435	CA		3.34
		Val435	C		3.76
		Phe436	N	H	3.35
Thr596	CG2	Asp434	O		3.79
		Val435	CG2		3.99
Asn597	CA	Phe436	N		3.95
		Phe436	O		3.76
Asn597	C	Phe436	O		3.95
Asn597	CB	Phe436	CB		3.87
		Phe436	O		3.82
Ser598	N	Phe436	O	H	3.17
Ser598	CB	Gln437	NE2		3.46
		Phe436	O		3.92
		Gln437	CD		3.91
		Gln437	OE1		3.88
Ser598	OG	Gln437	NE2		3.84
		Gln437	OE1		3.99
Glu599	C	Ser586	O		3.84
Glu599	O	Ser586	C		3.94
		Ser586	O		2.99
Glu599	CB	Ser586	O		3.77
Glu599	CD	Vak438	CG1		3.65
Glu599	OE1	Vak438	CB		3.58
		Vak438	CG1		3.32
		Ser586	CB		3.62
Glu599	OE2	Val438	N	H	3.39
		Val438	CB		3.78
		Val438	CG1		3.21

Asn600	CB	Gln587	O		3.92
Asn600	CG	Met585	O		3.85
Asn600	ND2	Met585	O	H	3.46
Ile628	CA	His419	ND1		3.51
Ile628	C	His419	ND1		3.18
		His419	CE1		3.56
		His419	CG		3.72
Ile628	O	His419	CB		3.35
		His419	ND1		3.06
		His419	CE1		3.69
		His419	CG		3.13
		His419	CD2		3.77
Ile628	CB	His419	ND1		3.28
		His419	CE1		3.52
His629	N	His419	ND1		3.84
		His419	CE1		3.76
His629	CB	His419	NE2		3.88

* H- hydrogen bond, S- salt bridges

Supplementary Table 2. **Oligonucleotide primers for site directed mutagenesis.**

Name	Sequence
HuMPEG L425K (fw)	5'- GGA AGT GTA CTT TGA AGG TGT TCT GC -3'
HuMPEG L425K (rev)	5'- GTC TTG CAG AAC ACC TTC AAA GTA C -3'

Supplementary Table 3. **Cryo-electron microscopy data collection, refinement and validation statistics.**

	MPEG1 WT		MPEG1α L425K			MPEG1β L425K	MPEG1 WT (liposome)	MPEG1 L425K (liposome)
**Data collection and processing**								
Magnification	128k ×		75k ×				105k ×	105k ×
Anisotropy (Angle, Major, Minor)	N/A		67°, 1.013, 1.000				N/A	N/A
Voltage (kV)	300		300				300	300
Electron exposure (e^{-} Å^{-2})	45		44.8				53.6	53.6
Exposure rate (e^{-} pix^{-1} s^{-1})	54.5		3.9				8	8
Exposure duration (s)	1		12.8				12	12
Set defocus range (μm)	−0.6 to −3.5		−0.7 to −2.5				−0.7 to −3.0	−0.7 to −3.0
Measured defocus (μ ± σ) (μm)	2.4 ± 1.0		1.7 ± 0.6				2.6 ± 1.2	1.8 ± 0.6
Camera	Falcon II		K2 Gatan Summit				K2 Gatan Summit	K2 Gatan Summit
Mode	Integration		Super Resolution				Counting	Counting
Frames	17		32				30	30
Pixel size (Å pix^{-1})	1.1		0.53				1.4	1.4
Micrographs	1,119		1,795				2,531	6,228
Particles (Total)	54,383	202,326^	133,631	267,262^	4,276,192^	133,631	12,364	38,479
Particles (Final)	25,359	202,326^	12,578	138,422^	527,402^	6,265	4,217	12,311
Reconstruction Strategy	Standard	LocalRec	Standard	LocalRec	LocalRec	Standard	Standard	Standard
Binning factor	1	1	2	2	2	2	1	1
Symmetry Imposed	D16	C16	D16	C16	C1	D16	C16	C16
Global Resolution (Å)								
0.5 (Unmasked / Masked)	4.16 / 3.70	4.00 / 3.21	3.96 / 3.36	3.42 / 2.75	3.89 / 3.29	3.85 / 3.26	12.0 / 10.8	4.20 / 3.76
0.143 (Unmasked / Masked)	3.93 / 3.49	3.03 / 2.91	3.39 / 3.01	2.98 / 2.37	3.46 / 2.93	3.29 / 2.83	9.55 / 8.94	4.01 / 3.63
Local resolution range (Å)#	> 4.4 to 3.1	> 4.0 to 2.8	> 4.2 to 2.7	> 4.0 to 2.2	> 3.9 to 2.9	> 4.2 to 2.6	> 12.0 to 7.0	> 4.5 to 3.2
Sphericity of 3DFSC	0.989	0.992	0.978	0.987	0.958	0.964	N/A	0.991
Map sharpening *B* factor (Å^{2})	−133	−72	−70	−78	−84	−57	−550	−145
EMDB identifier*	EMBD 20617	EMBD 20616	EMBD 20619	EMBD 20620	EMBD 20621	EMBD 20623	EMBD 20622	EMBD 20627

#As determined by windowed FSC at 0.143 criterion in Relion (2.1)

*Mask, half-maps, unsharpened map, locally-filtered sharpened map if applicable.

^Subparticles from localised reconstruction.

	MPEG1 WT		MPEG1α L425K			MPEG1β L425K	MPEG1 WT (liposome)	MPEG1 L425K (liposome)
**Refinement**								
Initial model (PDB code)	N/A	*de novo*	N/A	*de novo*	*de novo*	*de novo*	N/A	*de novo*
Model-to-map resolution								
CC (volume)		0.81		0.81	0.83	0.82		0.79
(Å, FSC 0.143 / 0.5)		2.9/3.0		2.2/2.4	2.8/3.1	2.6/2.8		3.2/3.9
B factors (Å^2)								
Protein		47.26		60.9	81.27	56.78		90.24
Ligand		83.66		75.76	107.99	70.85		1119.11
R.M.S. deviation								
Bond length (Å)		0.007		0.007	0.01	0.009		0.005
Bond angles (º)		0.931		0.895	0.989	1.031		1.142
Ramachandran plot								
Favoured (%)		92.67		95.19	90.3	92.92		88.06
Allowed (%)		7.33		4.81	9.7	7.08		11.94
Disallowed (%)		0		0.00	0	0.00		0
Cβ deviations (%)		0		0.00	0	0.00		0
Validation								
EM-Ringer Score		2.84		4.12	NA	3.92		1.59
MolProbity Score		1.55		1.53	1.67	1.56		1.76
Clash score		3		4.13	3.32	3.15		3.78
Poor rotamers (%)		0.39		0.41	0.19	0.19		0.84
PDB identifier		PDB 6U23		PDB 6U2J	PDB 6U2K	PDB 6U2L		PDB 6U2W

Supplementary References

1. Leung, C. *et al.* Real-time visualization of perforin nanopore assembly. *Nat. Nanotechnol.* **12,** 467–473 (2017).
2. Parsons, E. S. *et al.* Single-molecule kinetics of pore assembly by the membrane attack complex. *Nat. Commun.* **10,** (2019).
3. Winn, M. D. *et al.* Overview of the CCP4 suite and current developments. *Acta Crystallographica Section D: Biological Crystallography* **67,** 235–242 (2011).
4. Krissinel, E. & Henrick, K. Inference of Macromolecular Assemblies from Crystalline State. *J. Mol. Biol.* **372,** 774–797 (2007).
