# Supplementary material for: The cryo-EM structure of the acid activatable pore-forming immune effector Macrophage-expressed gene 1
Source: Nat Commun. 2019 Sep 19;10:4288. doi: 10.1038/s41467-019-12279-2 (PMC6753088; doi:10.1038/s41467-019-12279-2)
Supplement: Supplementary file 2 — Reporting Summary [file 41467_2019_12279_MOESM2_ESM.pdf]

nature research

Corresponding author(s): James C. Whisstock

Last updated by author(s): Aug 22, 2019

Reporting Summary

Nature Research wishes to improve the reproducibility of the work that we publish. This form provides structure for consistency and transparency in reporting. For further information on Nature Research policies, see Authors & Referees and the Editorial Policy Checklist.

Statistics

For all statistical analyses, confirm that the following items are present in the figure legend, table legend, main text, or Methods section.

n/a	Confirmed	
☐	☒	The exact sample size (n) for each experimental group/condition, given as a discrete number and unit of measurement
☐	☒	A statement on whether measurements were taken from distinct samples or whether the same sample was measured repeatedly
☒	☐	The statistical test(s) used AND whether they are one- or two-sided<br>*Only common tests should be described solely by name; describe more complex techniques in the Methods section.*
☒	☐	A description of all covariates tested
☐	☒	A description of any assumptions or corrections, such as tests of normality and adjustment for multiple comparisons
☒	☐	A full description of the statistical parameters including central tendency (e.g. means) or other basic estimates (e.g. regression coefficient) AND variation (e.g. standard deviation) or associated estimates of uncertainty (e.g. confidence intervals)
☒	☐	For null hypothesis testing, the test statistic (e.g. F, t, r) with confidence intervals, effect sizes, degrees of freedom and P value noted<br>*Give P values as exact values whenever suitable.*
☒	☐	For Bayesian analysis, information on the choice of priors and Markov chain Monte Carlo settings
☒	☐	For hierarchical and complex designs, identification of the appropriate level for tests and full reporting of outcomes
☒	☐	Estimates of effect sizes (e.g. Cohen's d, Pearson's r), indicating how they were calculated

Our web collection on statistics for biologists contains articles on many of the points above.

Software and code

Policy information about availability of computer code

Data collection	SerialEM, EPU
Data analysis	mag_distortion_estimate, MotionCor2, gautomatch, crYOLO (v1.1), cryoSPARC (v1, v2), CTFFIND4 (v4.1.10), RELION (v1.4,2.1,3.0), EMAN (v2.2), cisTEM (v1.0b), localised_reconstruction.py, MonoRes, LocalDeblur, blocfilt, 3DFSC, Segger, Chimera, ChimeraX, pymol, VMD, Phenix, EMRinger, MolProbity, namd2/MDFF

For manuscripts utilizing custom algorithms or software that are central to the research but not yet described in published literature, software must be made available to editors/reviewers. We strongly encourage code deposition in a community repository (e.g. GitHub). See the Nature Research guidelines for submitting code & software for further information.

Data

Policy information about availability of data

All manuscripts must include a data availability statement. This statement should provide the following information, where applicable:

- Accession codes, unique identifiers, or web links for publicly available datasets
- A list of figures that have associated raw data
- A description of any restrictions on data availability

Data Availability

Data supporting the findings of this manuscript are available from the corresponding authors upon reasonable request. A reporting summary for this Article is available as a Supplementary Information file.

The source data underlying Figs 1f, and Supplementary Figs 8b are provided as a Source Data file.

Cryo-EM maps and atomic models have been deposited in the Electron Microscopy Data Bank (EMDB) under accession codes EMBD 20616, EMBD 20617, EMBD

20619, EMBD 20620, EMBD 20621, EMBD 20622, EMBD 20623 and EMBD 20627. Each EMDB entry includes five maps: (1) the low-pass-filtered map without amplitude correction; (2) the low-pass-filtered map with amplitude correction by a negative B-factor shown in Extended Data Table 1; (3) the half maps without any post-processing such as low-pass-filtering or amplitude correction; and (4) the mask associated with refinement and sharpening. Coordinates are available from the RCSB Protein Data Bank under accession codes PDB 6U23, PDB 6U2J, PDB 6U2K, PDB 6U2L and PDB 6U2W.

Field-specific reporting

Please select the one below that is the best fit for your research. If you are not sure, read the appropriate sections before making your selection.

☒ Life sciences ☐ Behavioural & social sciences ☐ Ecological, evolutionary & environmental sciences

For a reference copy of the document with all sections, see nature.com/documents/nr-reporting-summary-flat.pdf

Life sciences study design

All studies must disclose on these points even when the disclosure is negative.

Sample size	No sample size calculations were performed, n=4 up to n=20 (or greater) were obtained. We observed excellent reproducibility between experiments and replicates indicated marginal variation.
Data exclusions	No data were excluded from analyses.
Replication	All attempts at replication were successful.
Randomization	Randomization is not relevant, data is collected in single sessions and analyzed computationally without human intervention.
Blinding	Blinding not relevant, analysis and collection of data did not require statistical interpretation and human bias is mitigated by established validation metrics.

Reporting for specific materials, systems and methods

We require information from authors about some types of materials, experimental systems and methods used in many studies. Here, indicate whether each material, system or method listed is relevant to your study. If you are not sure if a list item applies to your research, read the appropriate section before selecting a response.

Materials & experimental systems

n/a	Involved in the study	
☐	☒	Antibodies
☐	☒	Eukaryotic cell lines
☒	☐	Palaeontology
☒	☐	Animals and other organisms
☒	☐	Human research participants
☒	☐	Clinical data

Methods

n/a	Involved in the study	
☒	☐	ChIP-seq
☒	☐	Flow cytometry
☒	☐	MRI-based neuroimaging

Antibodies

Antibodies used	anti-6xHis-tag HRP-conjugated antibody
Validation	Any 6xHis tag can be used to validate antibody, molecular markers are his tagged and act as positive controls. Species independent.

Eukaryotic cell lines

Policy information about cell lines

Cell line source(s)	Commercial source (Invitrogen)
Authentication	Cell lines were used for recombinant protein production. No authentication performed.
Mycoplasma contamination	Cell lines were not tested for mycoplasma infection.
Commonly misidentified lines<br>(See ICLAC register)	N/A
